# Metal-non-tolerant ecotypes of ectomycorrhizal fungi can protect plants from cadmium pollution

**DOI:** 10.3389/fpls.2023.1301791

**Published:** 2023-12-06

**Authors:** Taoxiang Zhang, Wenbo Pang, Tianyi Yan, Panpan Zhang, Juan He, Christopher Rensing, Wenhao Yang, Chunlan Lian

**Affiliations:** ^1^International Joint Laboratory of Forest Symbiology, College of Forestry, Fujian Agriculture and Forestry University, Fuzhou, China; ^2^Key Laboratory of Soil Ecosystem Health and Regulation of Fujian Provincial University, College of Resources and Environment, Fujian Agriculture and Forestry University, Fuzhou, China; ^3^Asian Research Center for Bioresource and Environmental Sciences, Graduate School of Agricultural and Life Sciences, The University of Tokyo, Tokyo, Japan

**Keywords:** phytoremediation, *Cenococcum geophilum* (*C.geophilum*), cadmium (Cd), membership function method, *Pinus massoniana* (*P. massoniana*)

## Abstract

The application of mycorrhizal fungi as a bioaugmentation technology for phytoremediation of heavy metal (HM) contaminated soil has attracted widespread attention. In order to explore whether the adaptation of *Pinus massoniana* (*P. massoniana*) to metal polluted soil depends on the metal adaptation potential of their associated ectomycorrhizal fungi (ECMF), we evaluated the cadmium (Cd) tolerance of 10 ecotypes of *Cenococcum geophilum* (*C. geophilum*) through a membership function method, and *P. massoniana* seedlings were not (NM) or inoculated by Cd non-tolerant type (JaCg144), low-tolerant (JaCg32, JaCg151) and high-tolerant (JaCg205) isolates of *C. geophilum* were exposed to 0 and 100 mg·kg^-1^ for 3 months. The result showed that, each ecotype of *C. geophilum* significantly promoted the growth, photosynthesis and chlorophyll content, proline (Pro) content and the activity of peroxidase (POD) of *P. massoniana* seedlings, and decreased malonaldehyde (MDA) content and catalase (CAT) and superoxide dismutase (SOD) activity. The comprehensive evaluation D value of the tolerance to Cd stress showed that the order of the displaced Cd resistance of the four ecotypic mycorrhizal *P. massoniana* was: JaCg144 > JaCg151 > JaCg32 > JaCg205. Pearson correlation analysis showed that the Sig. value of the comprehensive evaluation (D) values of the strains and mycorrhizal seedlings was 0.077 > 0.05, indicating that the Cd tolerance of the the *C. geophilum* isolates did not affect its regulatory effect on the Cd tolerance of the host plant. JaCg144 and JaCg151 which are non-tolerant and low-tolerant ecotype significantly increased the Cd content in the shoots and roots by about 136.64-181.75% and 153.75-162.35%, indicating that JaCg144 and JaCg151 were able to effectively increase the enrichment of Cd from the soil to the root. Transcriptome results confirmed that *C. geophilum* increased the *P. massoniana* tolerance to Cd stress through promoting antioxidant enzyme activity, photosynthesis, and lipid and carbohydrate synthesis metabolism. The present study suggests that mental-non-tolerant ecotypes of ECMF can protect plants from Cd pollution, providing more feasible strategies for ectomycorrhizal-assisted phytoremediation.

## 1 Introduction

Heavy metal (HM) pollution has become one of the most serious environmental problems causing damage to water, air and soil, and posing potential risks to ecosystem (Zhou et al., 2008). Due to its poor stability and non-degradability, cadmium (Cd) is one of toxic heavy metals, which represents as potential threat to human health (Zhao et al., 2012; Yang et al., 2016). Meanwhile, Cd has been shown to be able to disturb the restriction of photosynthesis, induce of oxidative stress, and disrupt micro- and macro-elemental balance, leading to the destruction of forest ecosystems and ultimately affect human health (Chaudhary, 2017). Therefore, the restoration of Cd contaminated soil is critical for the protection of human and environmental health (Gong et al., 2018). Currently, many remediation technologies have been applied to the heavy metal polluted soils, including physical remediation, chemical remediation, and bioremediation (Liu et al., 2020). Phytoremediation is an economically effective remediation strategy with minimal environmental disturbance, which has received widespread attention in ecological environment restoration (Kushwaha et al., 2015). The remediative effect of phytoremediation on HM contaminated soil mainly depends on the extraction capacity and biomass of the employed plants (Oh et al., 2014), but most of the hyperaccumulating plants have some disadvantages such as being short plants, and displaying slow growth, low biomass, limited root expansion depth, and low transfer rate of HMs (Oladoye et al., 2022). Therefore, the key to promoting phytoremediation has been to improve plant biomass and HM transfer rate (Cundy et al., 2016; Pilipović et al., 2019). In previous studies, researchers have combined the measured biomass, chlorophyll content, osmoregulatory substance content and antioxidant enzyme activity with the membership function method to comprehensively evaluate the Cd tolerance of plants, which is critical for characterizing phytoremediation potential of plants (Tian et al., 2018; Huang et al., 2023). Inoculating mycorrhizal fungi to form mycorrhizal symbioses with plant roots has been shown to promote plant nutrient absorption and growth, enhance plant stress resistance under HM stress, and thus improve repair efficiency (Colpaert et al., 2011; Heinonsalo et al., 2015; Fernández-Fuego et al., 2017; Dagher et al., 2020). For instance, Xiao et al. (2023) found that *B. limosa* PY5 alleviated the phytotoxicity of Cd and enhanced poplar HM tolerance, resulting in increased plant growth. *P. tinctorius* and *C. geophilum* significantly improved the growth of pine shoots which planted in polluted soil (p< 0.01), and the Cu accumulated in pine seedlings increased by 72.8 and 113.3%, respectively, indicating that ectomycorrhizal fungi (ECMF) were able to help their host phytoextract HMs (Wen et al., 2017). Currently, the joint remediation of HM pollution in soil by plant-mycorrhizal fungi has attracted increasing attention (Huang et al., 2018; Dagher et al., 2020).

The use of rapidly growing trees with limited metal absorption capacity to restore contaminated soil and reduce pollution in metal polluted soil has previously been reported (Colpaert et al., 2011; Yu et al., 2020). Willows, poplars, birches and pines species have been able to build up pioneer populations, and shown great phytoremediation potential in HM-contaminated soils (Sell et al., 2005; Pilipović et al., 2019; Schat et al., 2020). For instance, poplars and willows have successfully been used for the phytoremediation of sediments, where Zn and Cd were mostly phytoextracted in leaves, and Cr, Ni, Pb and Cu were mostly phytostabilized in the roots of poplar and willow (Pilipović et al., 2019). In China, *Pinus massoniana* (*P. massoniana*) which is a fast-growing woody species with a large amount of biomass and a deep root system is widely distributed, and is still able to grow effectively under harsh environmental conditions, including barren, dry and polluted mines (Yu et al., 2020). Hence, *P. massoniana* is an important pioneer tree species in the construction and ecological restoration of severely HM polluted soil (Zhang et al., 2017; Pilipović et al., 2019). However, previous studies have shown that the adaptive potential of trees to metal tolerance is relatively low, and the colonization of trees in metal contaminated soil may be very slow (Meharg and Cairney, 1999). Even trees with high HM tolerance may take a long time to establish a significant tolerant population (Colpaert et al., 2011). Nevertheless, it has been believing that ECMF typically exhibits a higher tolerance against metal toxicity than plants, and ECMF has the ability to successfully colonize tree roots under polluted and barren soil conditions, effectively promoting plant tolerance to stress environments (Colpaert et al., 2011). *P. massoniana* are shown to be highly dependent on the presence of ECMF (Sousa et al., 2011; Wen et al., 2016). In HM polluted sites, *P. massoniana* were able to resist extreme metal toxicity through their association with some well-adapted ECMF, including dark ascomycetes and some metal-tolerant basidiomycetes (Chen et al., 2015; Zong et al., 2015). Hence, mycorrhizal fungi have been identified as one of the factors enabling *P. massoniana* to adapt to HM pollution (Huang et al., 2012).

ECMF has been shown to be symbiotic with many woody plants, promoting the absorption of water and nutrients for plants, improving plant growth under harsh conditions such as drought, salinization, and HM pollution, and enhancing tree colonization (Luo et al., 2014; Heinonsalo et al., 2015; Canton et al., 2016; Arraiano-Castilho et al., 2020; Dagher et al., 2020). *Hebeloma* sp., *Pisolithus* sp. (Zong et al., 2015), *Pisolithus tinctorius*, *Suillus* spp., *Cenococcum geophilum* (Goncalves et al., 2009; Wen et al., 2016), and *Laccaria* sp. (Liu et al., 2020) requently found on HM-polluted soils, and displayed adaptive HM tolerance. At present, most studies have mainly focused on the differences in metal tolerance between different fungal species, and few studies have considered intra-species differences among ECMF isolates from different locations and hosts in metal tolerance (Colpaert et al., 2011).

*Cenococcum geophilum* (*C. geophilum*) is one of the most common ECMF species with a wide variety of host species (Shi et al., 2022). Compared to other ECMF species, *C. geophilum* has a stronger adaptability to geographical environments in harsh habitats, and *C. geophilum* is one of the most suitable ECMF that has been shown to be capable to help in phytomediation of HMs (Colpaert et al., 2011; Wen et al., 2017). Liu et al. (2020) showed that after six months of planting, only 6.7% ± 14.1% of NM *P. densiflora* survived, while the survival rates of *P. densiflora* inoculated with *C. geophilum* significantly improved by 50%. Our previous research has shown that *C. geophilum* from different locations and hosts exhibited significant differences in tolerance to HM Cd (Shi et al., 2022). However, in a single species, different fungal ecotype may have evolved with the environment, exhibiting genetic adaptations that are beneficial for their own and host survival (Adriaensen et al., 2006). Hence, not all fungal ecotypes show ecotypic adaptation conducive to their own and host survival. Furthermore, most of the researches have mainly been focused on exploring how metal-tolerant ecotypes of ECMF protect plants from HM pollution? However, can metal-no-tolerant ecotypes protect plants from HM pollution, and what are the underlying mechanisms for their effects?

In this study, we inoculated *P. massoniana* with different fungal ecotypes of *C. geophilum* isolated from China and Japan. Our objectives were to identify the correlation between the Cd tolerance of the different fungal ecotypic *C. geophilum* strains and the inoculated *P. massoniana* seedlings, as well as the underlying mechanism of different ecotypes of *C. geophilum* on the Cd tolerance of *P. massoniana* which will provide more feasible strategies for ectomycorrhizal-assisted phytoremediation.

## 2 Materials and methods

### 2.1 Mycelial growth of different ecotype of *C. geophilum* strains under different cadmium treatments

Ten strains of *C. geophilum* (ChCg57, ChCg77, JaCg32, JaCg41, JaCg45, JaCg49, JaCg57, JaCg144, JaCg151and JaCg205) provided by the International Joint Laboratory of Forest Symbiology at Fujian Agriculture and Forestry University were used in this study, and the source and host information of each strain is shown in **Table S1**. The 10 ecotypic *C. geophilum* strains were cultured in modified Melin-Norkrans (MMN) agar medium at 25°C for 45 days. 7 mm diameter agar plugs were transferred to the centre of each petri dish containing 20 ml MMN agar medium with different Cd concentrations (0, 2, 4, 6 and 8 mg/L CdCl_2_ ·2.5H_2_O), and cultured in the dark at 25°C for 30 days. Each strain was replicated three times for each treatment. After 30 days in culture, the mycelial area was measured using a planimeter (X-Plan 380dIII, Kantum, Yokohama, Japan).

### 2.2 Evaluation of cadmium tolerance of different ecotype of *C. geophilum* strains and *P. massoniana* mycorrhizal seedlings

The Cd resistance coefficient (CRC) was calculated to study the tolerance of different ecotypes of *C. geophilum* and *P. massoniana* mycorrhizal seedlings to Cd stress. The CRC was calculated by:


CRC=VinVic×100

where Vin and Vic correspond to the trait values in the stressed treatment and control, respectively (Zou et al., 2020).

The D value which present the Cd tolerance level of different ecotypic *C. geophilum* and *P. massoniana* mycorrhizal seedlings were subsequently calculated (Zou et al., 2020).

Calculate the membership function values (U_i_) of various indicators for strains and *P. massoniana* mycorrhizal seedlings by the following formula:


(1) Ui=(Xi−Xmin)(Xmax−Xmin)

where U_i_ was the value of the membership function of the index i, X_i_ was the value of the index i, and Xmin and Xmax were the minimum and maximum values of j index, respectively.

Based on the PCA, the weight (Wi) have been calculated on the basis of the contribution rate (Y) of each index:


(2) Wi=PiΣi=1nPi

where W_i_ represented the weight of the comprehensive index in all the comprehensive indices, the weight of pi represented the contribution rate of each *P. massoniana* mycorrhizal seedlings to the comprehensive index.

The D value of the final comprehensive evaluation of the tolerance level to Cd stress of different ecotypic *C. geophilum* and *P. massoniana* mycorrhizal seedlings:


(3) $$\text{D}=\frac{1}{\text{n}}\Sigma_{i=1}^{n}U_{i}$$

(4) $$\text{D}=\Sigma_{i=1}^{n}(W_{i}\times U_{i})$$

Formula (3) was adapted for *C. geophilum* and formula (4) was adapted for *P. massoniana* mycorrhizal seedlings. The higher the D value, the greater Cd tolerance of *C. geophilum* and *P. massoniana* mycorrhizal seedlings.

To divide into tolerance type of different ecotypic *C. geophilum*, the Euclidean distance square and system clustering was used to cluster D values with an distance of 8 (Zou et al., 2020).

### 2.3 Preparation of ectomycorrhizal seedlings and experimental setup

According to the evaluation of Cd tolerance of *C. geophilum* strains, JaCg144 (non-tolerant type), JaCg151 (low-tolerant type), JaCg32 (high-tolerant type) and JaCg205 (high-tolerant type) were chosen in this experiment. *P. massoniana* seeds were provided by Wuyi National Forest Farm (Fujian, China). The seeds were sterilized with 1% NaClO for 10 minutes, soaked in sterile water for 24-48 hours, and sown in sterilized vermiculite (121°C, 3 hours). When the needles of *P. massoniana* were unfolding, the seeding of *P. massoniana* were moved to a rectangular Petri dish with sterilized substrate (Shibanome: forest soil=2:1) (Quan et al., 2023), and incubated for 15 days. The lateral roots of seedlings were inoculated by applying equal-sized fungal plugs which had been incubated for one month, and non-mycorrhizal seedlings (NM) of *P. massoniana* were used as control plants. The NM and mycorrhizal *P. massoniana* seedlings were cultured in a greenhouse with relative humidity ranging from 65% to 70% and 8-h dark (20°C)/16-h light (26°C) for 60 days to format mycorrhizae. The photosynthetic photon flux density was 300μmol m^–2^s^–1^ and 200 mL sterilized water was regularly irrigated once a week during cultivation. A stereomicroscope (OLYMPUS-S261, Olympus, Japan) was used to observe the mycorrhiza infection rate after 2 months of inoculation, and the Cd stress culture experiment of mycorrhiza seedlings were conducted when the infection rate reached 80% (**Figure S1**).

Forest soil (0-20 cm) from the cultivation layer in Fuzhou (China) was collected for pot experiment. The soil properties in the planting experiment are as follows: pH value is 5.92, organic matter content is 0.48 g·kg^-1^, total phosphorus content is 120 mg·kg^-1^, and total potassium content is 16.69 g·kg^-1^. Soil mixed with the Shibanome: at a ratio of 2:1, and treated with CdCl_2_·2.5H_2_O to achieve a final Cd concentration of 100 mg·kg^-1^, which keep equilibrated for 15 days. Finally, the culture medium autoclaved at 121°C for 3 h. Each culture cup (bottom diameter 5.2 cm, height 9.5 cm, volume 500mL) was prepared with 400 g of culture medium, and three uniform plants were selected and transferred to each pot. Each treatment contained 5 pots, 3 seedlings each pot, 3 seedlings pooled as a repeat, repeated 3 times. After 90 days’ growth in the temperature controlled greenhouse, plants were harvested for analysis of morphological, physiological and molecular characteristics.

### 2.4 Photosynthesis measurement and root morphology scanning

A Li-6400 portable photosynthetic instrument (GFS3000F, WALZ Corporation, US) was used to measure the net photosynthetic rate (Pn), transpiration rate (Tr), intercellular CO_2_ concentration (Ci) and stomatal conductance (Gs) of the needle leaves of each seedlings of *P. massoniana*. The measurement conditions were as follow: the CO_2_ concentration was 400 ppm, the relative humidity was 40-60% and the temperature was 24-26°C. The instrument setting was as follows: a red and blue light source were conducted under strong illumination (1200 μmE m^-2^ s^-1^). After adapting for a period of time, the light intensity was adjusting to 1000 μmE·m^-2^·s^-1^. non-mycorrhizal *P.massoniana* seedlings (NM) as the control.

After harvest, the redundant soil was shake off at the root, and the plants were thoroughly rinsed with deionized water. The EPSON scanner (Expression 11000XL, Seiko Epson Corporation, Japan) was used to scan the complete root image and Win RHIZO software (Regent, Quebec, Canada) was conducted to analyze root morphological indices, including root tips, average root diameter, root surface area, total root diameter and root volume. Three replicated *P.massoniana* seedlings were analyzed per treatment.

### 2.5 Determination of cadmium concentrations

The harvested *P. massoniana* seedlings were divided into shoots and roots, and dried to constant weight at 80°C. Plants were then grinded and sieved to <1 mm size. 0.1g of dried shoot and root power were weighed, and digested with 2.5 mL of mixed solution of concentrated 65% nitric acid (HNO_3_) (Xilong Chemical Co., Ltd., Guangdong) and perchloric acid (HClO_4_) (Kelong Chemical Factory, Chengdu, China) (v/v, 5:1) for 1.5 h in an automatic digester (ETHOS UP, Milestone, China). After diluting and filtering, the filtrate was used to determined Cd concentrations by inductively coupled plasma-atomicemission spectroscopy (ICP-AES, PerkinElmer NexION300X). To evaluate the efficiency of the phytoextraction of *P. massoniana* seedlings, the transfer factor (TF) and the bioconcentration factor (BF) were calculated. The formula is as follows:


$$\text{Transfer factor }\left(\text{TF}\right)\;=\text{ shoot Cd concentration }\left(\text{mg}\cdot \text{k}{\text{g}}^{-1}\right)\;/\text{ root Cd concentration }\left(\text{mg}\cdot \text{k}{\text{g}}^{-1}\right);$$

$$\text{Bio− concentration factor }\left(\text{BF}\right)\;=\text{ shoot Cd concentration }\left(\text{mg}\cdot \text{k}{\text{g}}^{-1}\right)\;/\text{ soil Cd concentration }\left(\text{mg}\cdot \text{k}{\text{g}}^{-1}\right).$$

### 2.6 Analysis of photosynthetic pigment, proline and malonaldehyde content and antioxidant enzyme activities

For the analysis of chlorophylls (chlorophyll-*b* and Chlorophyll-*a*), 0.1g of fresh shoot and root samples were homogenized with 5 ml of 80% acetone. The absorbances of the supernatant were measured at 470, 649 and 664 nm using a microplate reader (SpectraMax ID5, Molecular Devices, USA), and the photosynthetic pigment contents were calculated (Li et al., 2022). Malonaldehyde (MDA), proline (Pro), and antioxidant enzymes were assayed using 0.1 g samples according to the instructions provided by the kit manufacturer (Suzhou Comin Biotechnology Co., Ltd. Suzhou, China).

### 2.7 RNA-Seq analysis and screen of differentially expressed genes

The total RNA of *P. massoniana* roots was extracted by Concert Plant RNA Reagent (Invitrogen, USA). The integrity and concentration of the obtained RNA was checked using a Nano Drop 2000 UV–vis Spectrophotometer (Thermo Fisher Scientific, Waltham, MA, USA) and RNase-free agarose gel electrophoresis (2%) to ensure that all samples met the requirements for library construction for further analysis. The cDNA library was constructed, quality verified, and sequenced by Biomarker Technologies (Beijing, China) using an Illumina NovaSeq 6000 platform according to standard protocols. After filtering the original sequencing data, the error rate and GC-content distribution were checked to obtain high-quality clean reads.

The BLAST program was used to annotate the unigenes, with an Evalue threshold of 1e-5 in the NR, Swiss Prot, COG, KOG, egg NOG, KEGG, Pfam, and TrEMBL databases. Gene expression was normalized to RPKM. Differential expression analysis was performed by DEGseq2 software, with fold change ≥ 2 and false discovery rate ≤ 0.01 as criteria for screening differential genes, and then subjected to enrichment analysis using the GO functions and KEGG pathways.

### 2.8 Validation by RT-qPCR

Ten differentially expressed genes were selected for validation. The 2^−ΔΔCt^ method was used to calculate the relative gene expression of RT-qPCR. The gene *GAPDH* (Glyceraldehyde 3-Phosphatase) was used as an internal control. Premier 5.0 (http://www.premierbiosoft.com) was used to design specific primer sequences for selected genes (**Table S2**).

### 2.9 Statistical analysis

Statistical analysis was performed using SPSS (Statistical Product and Service Solutions 21.0) software. The analysis of the principal components and the cluster analysis were evaluated using the IBM SPSS statistics package (version 24.0) (IBM Inc., Ammonk, N.Y., USA). One-way analysis of variance (ANOVA) and Duncan’s test were used to evaluate the data at a significance level of *p*< 0.05 under the condition of satisfying normality and homogeneity of variance. Data were presented as mean ± SE of replicates (n=3). Data visualization was performed using Origin 2022.

## 3 Results

### 3.1 Tolerance of different ecotypes of *C. geophilum* to Cd stress

**Figure 1:**
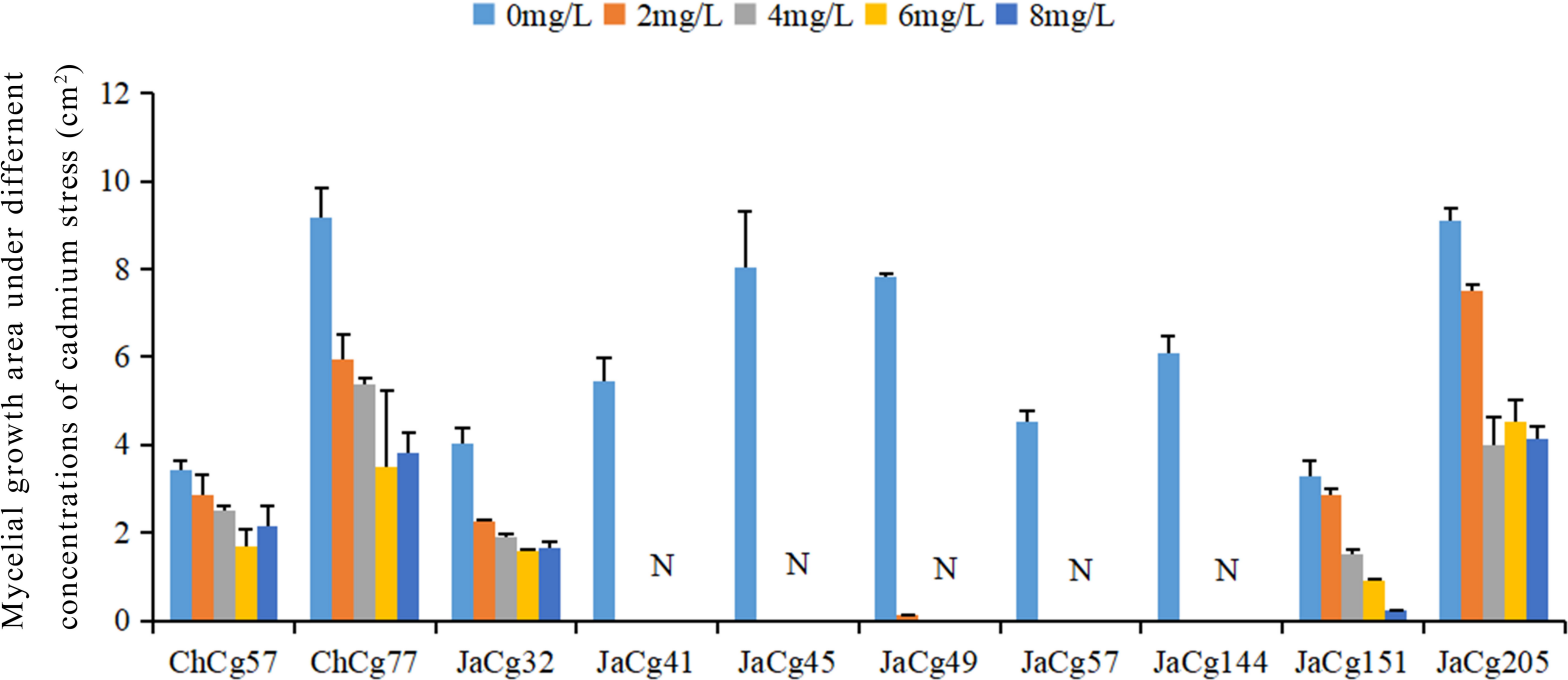
The growth area of the ten strains of *C. geophilum* under different Cd concentrations. Data and bars are shown as mean and ± SE of the replicates, respectively (n = 3). N represents that the strain did not grow.

Ten strains of *C. geophilum* were cultured under 0, 2, 4, 6, and 8 mg·L^-1^ Cd stress (**Figure 1**). In the absence of Cd, there were difference in growth area between different ecotypes of *C. geophilum*. The growth area of ChCg77 was the largest, followed by JaCg205, JaCg49, JaCg144, JaCg41, JaCg32, ChCg57, and JaCg151. However, the growth of each ecotype of *C. geophilum* was significantly inhibited by elevated Cd levels in the medium, and colony area of them were decreased by 37.32-100% when the colonies were exposed to 0-8 mg·L^-1^ Cd for four weeks. Among them, JaCg41, JaCg45, ChCg57, and JaCg144 displayed a colony area of 0 when the Cd concentration was 2 mg·L^-1^, and none of these strains showed any signs of growth with increasing Cd concentrations, indicating that these strains were unable to adapt to Cd stress. The growth area of other ecotypic strains generally showed a gradually decreasing trend, but there were slight differences in the changing trend among different ecotypic *C. geophilum* strains. For example, the colony area of ChCg77 and JaCg205 gradually remained constant after treatment with 4 and 6 mg·L^-1^ Cd. The strain area of Chcg57 and Jacg32 was decreased by 17.20% and 44.03%, when exposed to 2 mg·L^-1^ Cd. However, 2-8 mg·L^-1^ Cd did not affect the growth of their colony area.

### 3.2 Evaluation of Cd tolerance of *C. geophilum* strains

**Figure 2:**
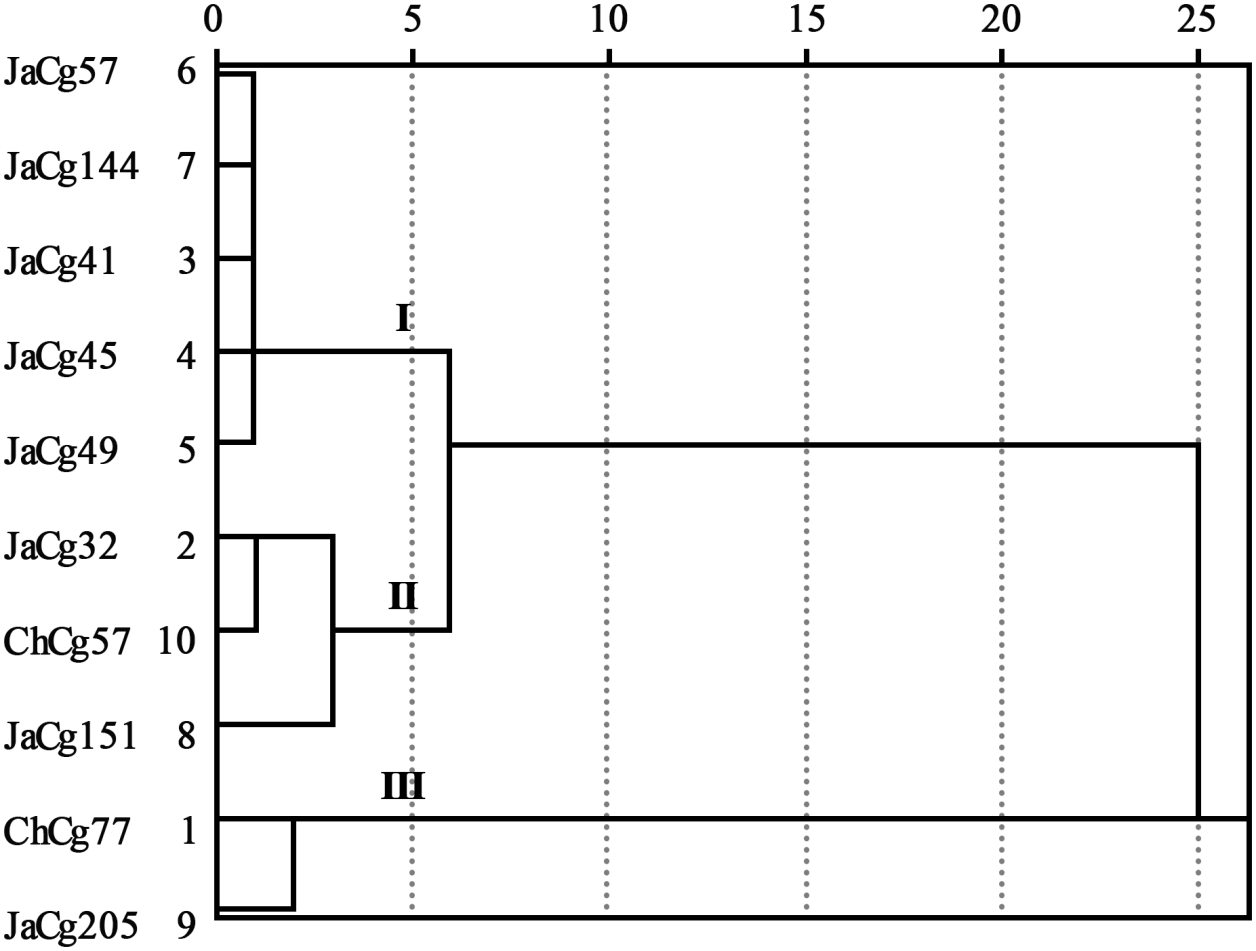
The dendrogram of clusters for the ten strains of *C. geophilum*.

According to formulas (3) and (4), the membership function values (D) of various indicators of the strains were calculated. The results indicated that the order of the resistance to Cd stress of 10 different ecotypes of *C. geophilum* was: JaCg205 > ChCg77 > ChCg57 > JaCg32 > JaCg151 > JaCg49 > JaCg41 > JaCg45 > JaCg57 > JaCg144 (**Table 1**). By the cluster analysis of the membership function values (D), 10 different ecotypic types of *C. geophilum* were divided into 3 categories (**Figure 2**): JaCg57, JaCg144, JaCg41, JaCg45, and JaCg49 are classified as Class I, belonging to Cd non-tolerant type; JaCg 32, ChCg57, and JaCg151 are classified as Class II and belong to the low-tolerance type; ChCg 77 and JaCg 205 are classified as Class III and belong to the high-tolerance type.

**Table 1:** Ranking of resistance to Cd stress of *C. geophilum* strains.

Code	D value	Sort
JaCg205	0.935	1
ChCg77	0.871	2
ChCg57	0.487	3
JaCg32	0.351	4
JaCg151	0.228	5
JaCg49	0.004	6
JaCg41	0	7
JaCg45	0	8
JaCg57	0	9
JaCg144	0	10

### 3.3 The biomass and root structure of mycorrhizal *P. massoniana* seedlings with different ecotypes of *C. geophilum*

**Figure 3:**
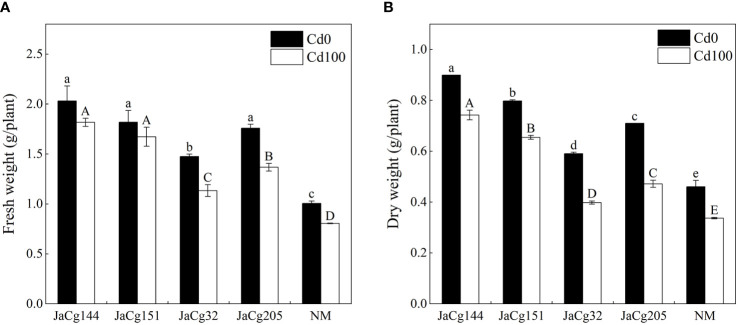
Fresh weight **(A)** and dry weight **(B)** of non-inoculated (NM) and inoculated (JaCg144, JaCg151, JaCg32 and JaCg205) *P. massoniana* plants under Cd stress. Data and bars are shown as mean and ± SE of the replicates, respectively (n = 3). Different letters indicate statistically significant differences between different ecotype of *C*. *geophilum* inoculations using one-way ANOVA followed by Dunnett’s test (P<0.05).

**Figure 3** shows the effects of Cd stress on the fresh weight and dry weight of inoculated and non-inoculated *P. massoniana* seedings (**Figures 3A, B**). When the soil Cd concentration was 0 and 100 mg·kg^-1^, *C. geophilum* significantly promoted the growth of *P. massoniana* seedlings. However, when compared with the treatment without Cd addition, Cd stress caused a significant decline in the fresh and dry weight of *P. massoniana* seedings (*P*<0.05). Four ecotypes of *C. geophilum* significantly increased the fresh and dry weight of *P. massoniana* seedings at 100 mg·kg^-1^ Cd. The biomass of the mycorrhizal seedlings was higher than that of NM, with the fresh weight being 1-1.96 times of NM, and the dry weight being 1-2.2 times of NM, indicating that *C. geophilum* promoted the resistance of *P. massoniana* to Cd. Above all, JaCg144 and JaCg151 which were non-tolerant and low-tolerant ecotypic strains displayed the strongest promoting role for plant growth.

**Figure 4:**
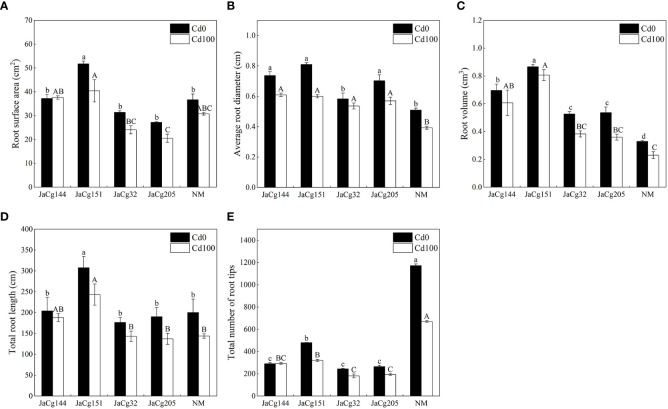
Root surface area **(A)**, average root diameter **(B)**, root volume **(C)** and total root diameter **(D)** and root tips **(E)** of non-inoculated (NM) and inoculated (JaCg144, JaCg151, JaCg32 and JaCg205) *P. massoniana* plants under Cd stress. Data and bars are shown as mean and ± SE of the replicates, respectively (n = 3). Different letters indicate statistically significant difference between different ecotype of *C*. *geophilum* inoculations using one-way ANOVA followed by Dunnett’s test (P<0.05).

**Figure 4** shows the root parameters of different ecotypes of *C. geophilum* seedlings. Cd stress inhibited the root development of *P. massoniana* seedlings, but the phenomena was alleviated by the inoculation of *C. geophilum*. Under exposure to 100 mg·kg^-1^ Cd, the root surface area, average root diameter, root volume and root diameter of each ecotypic mycorrhiza seedlings were higher than those of NM group, especially there were significant differences in root volume and root diameter. Among the four inoculation treatments, no difference was found in the root structure pattern. Interestingly, the number of root tips of *P. massoniana* seedlings was significantly inhibited by *C. geophilum*. Compared with NM, the number of root tips of *P. massoniana* seedlings inoculated with *C. geophilum* was reduced by 56.19%, 65.49%, 73.00%, and 71.06%, respectively.

### 3.4 Photosynthetic parameters and chlorophyll content of mycorrhizal *P. massoniana* seedlings with different ecotypes of *C. geophilum*

**Figure 5:**
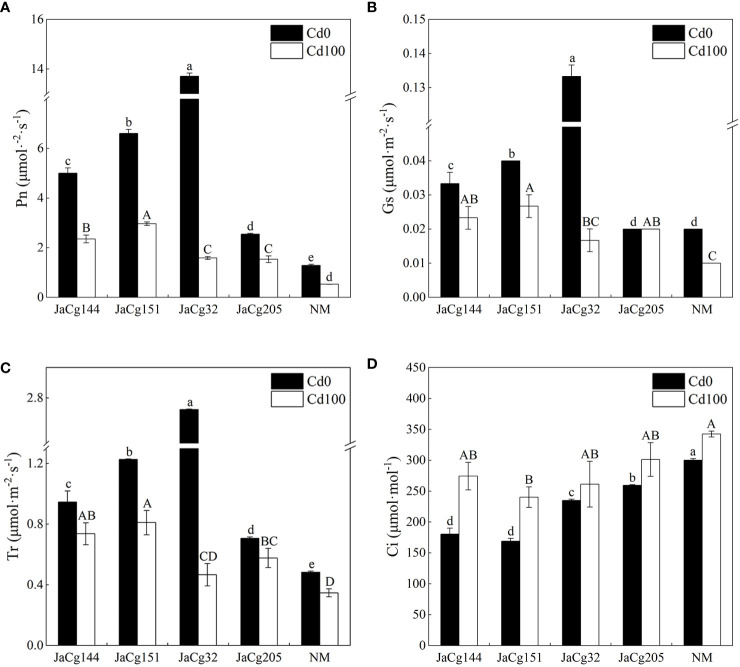
Net photosynthetic rate (Pn) **(A)**, stomatal conductance (Gs) **(B)**, transpiration rate (Tr) **(C)**, and intercellular CO_2_ (Ci) **(D)** of non-inoculated (NM) and inoculated (JaCg144, JaCg151, JaCg32 and JaCg205) *P. massoniana* plants under Cd stress. Data and bars are shown as mean and ± SE of the replicates, respectively (n = 3). Different letters indicate statistically significant difference between different ecotype of *C*. *geophilum* inoculations using one-way ANOVA followed by Dunnett’s test (P<0.05).

In the absence of Cd stress, 4 ecotypes of *C. geophilum* displayed a significant role in promoting photosynthesis of *P. massoniana* seedlings (**Figure 5**). Especially for strain JaCg32, compared to NM, the Pn, Gs, and Tr of mycorrhizal seedlings increased by 90.59%, 85.00%, and 82.25%, and the Ci significantly decreased. However, this promoting effect was weakened by Cd stress. Cd treatment significantly inhibited the photosynthesis of mycorrhizal seedlings and non-mycorrhizal seedlings. However, the Pn, Gs, and Tr of mycorrhizal seedlings were higher than those of NM.

**Figure 6:**
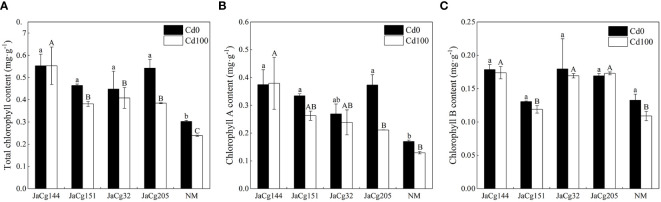
Total chlorophyll content **(A)**, chlorophyll *a* content **(B)**, and chlorophyll *b* content **(C)** of non-inoculated (NM) and inoculated (JaCg144, JaCg151, JaCg32 and JaCg205) *P. massoniana* plants under Cd stress. Data and bars are shown as mean and ± SE of the replicates, respectively (n = 3). Different letters indicate statistically significant difference between different ecotype of *C*. *geophilum* inoculations using one-way ANOVA followed by Dunnett’s test (P<0.05).

**Figure 6** shows the effects of different ecotypes of *C. geophilum* on the chlorophyll content of *P. massoniana* seedlings under Cd stress. In the absence of Cd stress, compared with NM, the strains of each ecotype significantly increased the total chlorophyll content of *P. massoniana* seedlings (48.24% -79.29%). Among these ecotypes, the strain JaCg144 had the greatest impact on the chlorophyll content of *P. massoniana* seedlings, as the contents of chlorophyll *a*, Chlorophyll *b* and total chlorophyll increased by 120.39%, 34.67% and 82.82%, respectively. Under Cd stress, the chlorophyll *a*, chlorophyll *b*, and total chlorophyll content of *P. massoniana* were decreased, but there was no significant effect on the chlorophyll content of JaCg144 seedlings. Compared to the NM, the total chlorophyll content of JaCg 144, JaCg 151, JaCg 32, and JaCg 205 mycorrhizal seedlings increased by 132.35%, 60.37%, 71.57%, and 61.63%, respectively.

### 3.5 The content of MDA and Pro content, and antioxidant enzyme activities of *P. massoniana* seedlings

**Figure 7:**
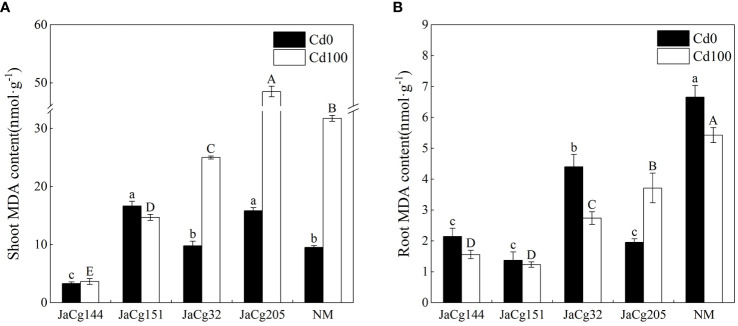
Malondialdehyde (MDA) content in the shoot **(A)** and root **(B)** of non-inoculated (NM) and inoculated (JaCg144, JaCg151, JaCg32 and JaCg205) *P. massoniana* plants under Cd stress. Data and bars are shown as mean and ± SE of the replicates, respectively (n = 3). Different letters indicate statistically significant differences between different ecotype of *C*. *geophilum* inoculations using one-way ANOVA followed by Dunnett’s test (P<0.05).

As shown in **Figure 7**, the contents of MDA in the root and shoot were used to evaluate the oxidative stress response of *P. massoniana* seedlings under Cd stress. An increase in MDA content was observed in shoots of both NM and mycorrhizal plants with 100 mg·kg^- 1^ Cd in soils. The content of MDA in the shoots was higher than that in the roots of *P. massoniana* seedlings, and *C. geophilum* decreased the accumulation of MDA in the shoot and roots. The MDA content in the roots and shoots of JaCg144 and JaCg151 were significantly lower than those of JaCg32 and Jacg205. In the presence of 100 mg·kg^- 1^ Cd, mycorrhizal seedlings JaCg144 displayed the lowest shoot MDA content, with 11.52% of NM (**Figure 7A**), while mycorrhizal seedlings of JaCg151 were observed the lowest root MDA content, with 22.71% of NM (**Figure 7B**).

**Figure 8:**
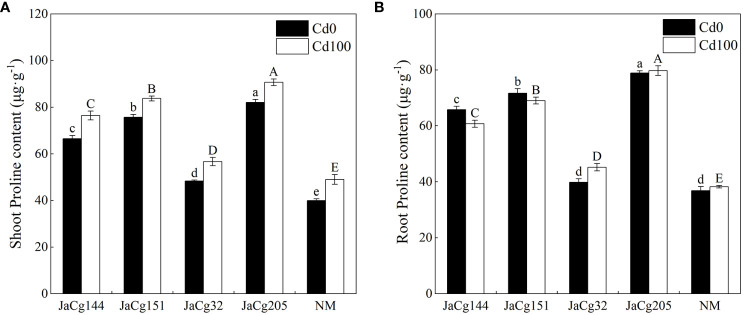
The proline (Pro) content in the shoot **(A)** and root **(B)** of non-inoculated (NM) and inoculated (JaCg144, JaCg151, JaCg32 and JaCg205) *P. massoniana* plants under Cd stress. Data and bars are shown as mean and ± SE of the replicates, respectively (n = 3). Different letters indicate statistically significant difference between different ecotype of *C*. *geophilum* inoculations using one-way ANOVA followed by Dunnett’s test (P<0.05).

Under Cd levels of 100 mg·kg^-1^, the Pro content in the shoot of NM and mycorrhizal *P. massoniana* seedlings showed an increasing trend, while the Pro content in the root showed no significant change. Our results clearly indicated that *C. geophilum* significantly promoted the biosynthesis of Pro in *P. massoniana* seedlings in response to 100 mg·kg^-1^ Cd (**Figure 8**). The stimulatory effect was more pronounced in the shoot and root of plants inoculated with JaCg144, JaCg151 and JaCg205 by 55.89%, 58.85%, 70.80% and 80.58%, 85.02% and 108.59%, respectively, as compared to the NM.

**Figure 9:**
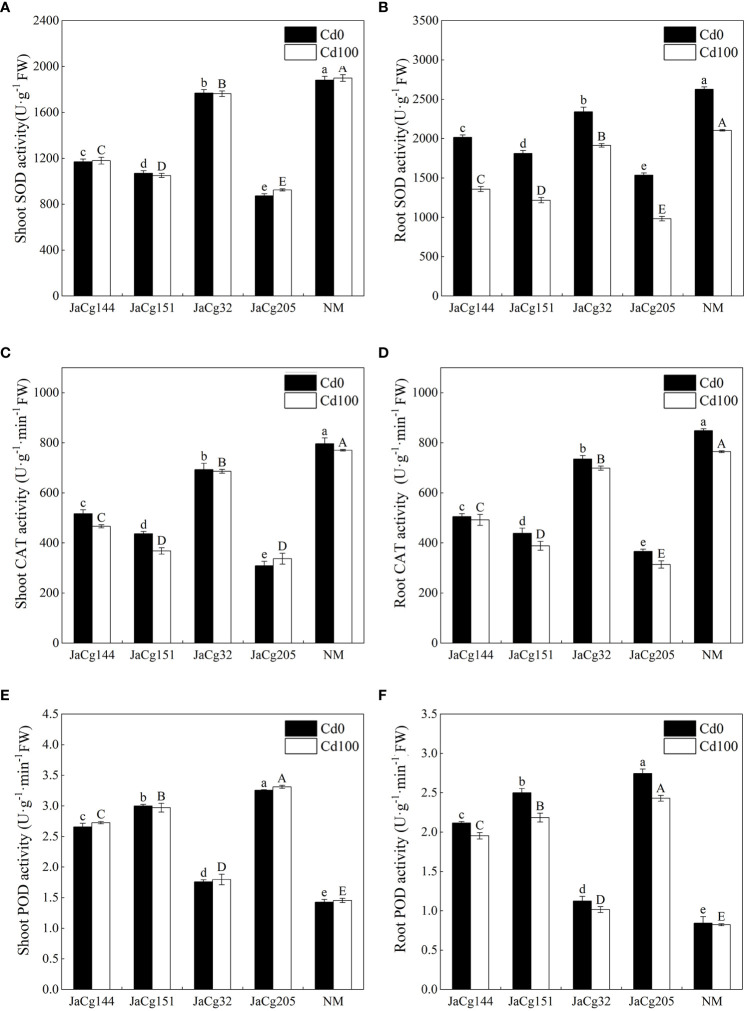
The antioxidant enzyme activities of non-inoculated (NM) and inoculated (JaCg144, JaCg151, JaCg32 and JaCg205) *P. massoniana* plants under Cd stress. Superoxide dismutase (SOD) activity in shoot **(A)** and root **(B)**, antio Catalase (CAT) activity in shoot **(C)** and root **(D)**, Peroxidase (POD) activity in shoot **(E)** and root **(F)**. Data and bars are shown as mean and ± SE of the replicates, respectively (n = 3). Different letters indicate statistically significant differences between different ecotype of *C*. *geophilum* inoculations using one-way ANOVA followed by Dunnett’s test (P<0.05).

**Figure 9** reflects the response of antioxidant enzyme activity of 4 ecotypes of mycorrhizal seedlings to Cd stress. Cd stress reduced the activity of SOD, CAT, and POD in the root of *P. massoniana* seedlings, but there was no significant change in the shoot. Compared to the control group NM, each ecotypic strain significantly reduced the activities of SOD and CAT in the shoot and root of *P. massoniana* seedlings, but significantly increased the activity of POD. JaCg 205 had the most significant effect on the activities of SOD, CAT, and POD in *P. massoniana* seedlings, with the SOD activity in shoot and root decreasing by 51.30% and 53.30%, and the CAT activity decreasing by 56.22% and 58.92%, respectively, while the POD activity increased by 127.45% and 195.24%.

### 3.6 Evaluation of Cd tolerance of mycorrhizal *P. massoniana* seedlings

After standardizing the Cd resistance coefficients of various evaluation indicators for Cd stress resistance (**Table S3**), principal component analysis was conducted. The cumulative contribution rate of the first three principal components reached 100% (**Table S4**), and then the comprehensive evaluation D value of the tolerance ability of different mycorrhizal *P. massoniana* to Cd stress was calculated. The results showed that the order of Cd resistance of four different ecotypes of mycorrhizal *P. massoniana* was: JaCg144 > JaCg 151 > JaCg32 > JaCg205 (**Table 2**). It was worth noting that, JaCg144 with the weakest Cd tolerance displayed the strongest promoting ability on the Cd tolerance of *P. massoniana*. Meanwhile, Pearson correlation analysis was conducted on the comprehensive evaluation (D) values of the strains and mycorrhizal seedlings, and the results showed that the Sig. value of the two was 0.077 > 0.05 (**Table 3**). The correlation between the two was not significant, indicating that the Cd tolerance of the strain did not affect its promoting effect on the Cd tolerance of the host plant.

**Table 2:** The comprehensive evaluation of the tolerance level to Cd stress of different *P. massoniana* mycorrhizal seedlings.

code	Comprehensive index			membership function			D	order
	F_1_	F_2_	F_3_	U_1_	U_2_	U_3_		
JaCg144	2.759	0.892	2.313	1.000	0.666	1.000	0.894	1
JaCg151	2.318	-0.756	-2.577	0.920	0.381	0.000	0.539	2
JaCg32	-2.722	2.821	-0.560	0.000	1.000	0.412	0.411	3
JaCg205	-2.355	-2.957	0.824	0.067	0.000	0.695	0.189	4
Weight				0.455	0.317	0.228		

**Table 3:** Correlation coefficient between comprehensive evaluation (D) values of the *C. geophilum* strains and *P. massoniana* mycorrhizal seedlings.

	D value of the strains	D value of mycorrhizal seedlings
D value of the strains	1	-0.923
D value of mycorrhizal seedlings	-0.923	1
Significance (p)	0.077	

### 3.7 Accumulation and translocation of Cd of *P.massoniana* seedlings

**Figure 10:**
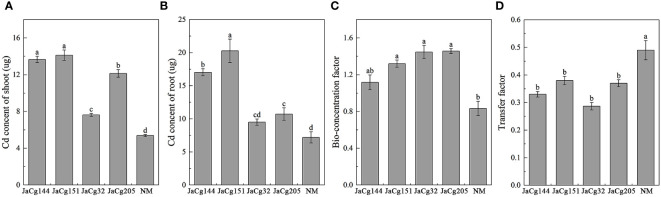
Cd content in shoot **(A)** and root **(B)**, bio-concentration factor **(C)** and transfer factor **(D)** of non-inoculated (NM) and inoculated (JaCg144, JaCg151, JaCg32 and JaCg205) *P. massoniana* plants under Cd stress. Data and bars are shown as mean and ± SE of the replicates, respectively (n = 3). Different letters indicate statistically significant difference between different ecotype of *C. geophilum* inoculations using one-way ANOVA followed by Dunnett’s test (P<0.05).

**Figure 10** reflects the migration and accumulation of Cd in the roots and shoots of *P. massoniana* seedlings after being cultured in Cd contained soil. Compared to NM plants, *C. geophilum* significantly increased the content of Cd in the shoots and roots in the four mycorrhizal plants in the presence of 100 mg·kg^-1^ Cd. For example, JaCg144 and JaCg151 significantly increased the content of Cd in the shoots and roots by about 136.64-181.75% and 153.75-162.35%. Though the content of Cd in shoots and roots of JaCg32 and JaCg205 was lower than JaCg144 and JaCg151, they were still significantly higher than in the NM seedling. JaCg144, JaCg151, JaCg32, and JaCg205 treatments significantly increased the BF of Cd by 34.01%, 58.41%, 73.61 and 74.81%, and significantly reduced the TF of *P. massoniana* (*p*<0.05) by 32.65%, 22.45%, 41.49% and 24.49%, respectively, indicating that *C. geophilum* was able to effectively increase the enrichment of Cd from the soil to the root, and reduce the transport of Cd from root to the shoot.

### 3.8 Differential gene expression under cadmium stress as determined by RNASeq

RNA-seq technology was applied to assay the gene expression after NM and mycorrhizal *P. massoniana* seedlings grown with 100 mg·kg^-1^ of Cd to better understand the remediation mechanism of *C. geophilum*. Detailed information for the 12 samples (three replicates for each sample) is listed in **Table S5**. On the whole, an average of 65170833 high-quality Clean reads were obtained and assembled into 58,106 unigenes as the reference transcripts. The average length was 1552.06 bp, and the N50 of the unigenes was 1882bp, suggesting high quality assembled RNA-Seq data (**Table S6**).

The differentially expressed genes (DEGs) of mycorrhizal seedlings under Cd0 and Cd100 treatments were compared (**Figure S2**). A total of 27506 DEGs were obtained, of which 4237 were up-regulated and 4739 were down-regulated in JaCg144, 542 were up-regulated and 1499 were down-regulated in JaCg151, 5170 were up-regulated and 6512 were down-regulated in JaCg32, and 2458 were up-regulated and 2349 were down-regulated in JaCg205. The Venn plot shows that 67 DEGs were up-regulated and 129 DEGs were down-regulated in all four mycorrhizal seedlings (**Figure S3**). Among them, 148 DEGs were up-regulated and 367 DEGs were down-regulated in the root seedlings of tolerant bacteria JaCg32, JaCg151, and JaCg144.

**Figure 11:**
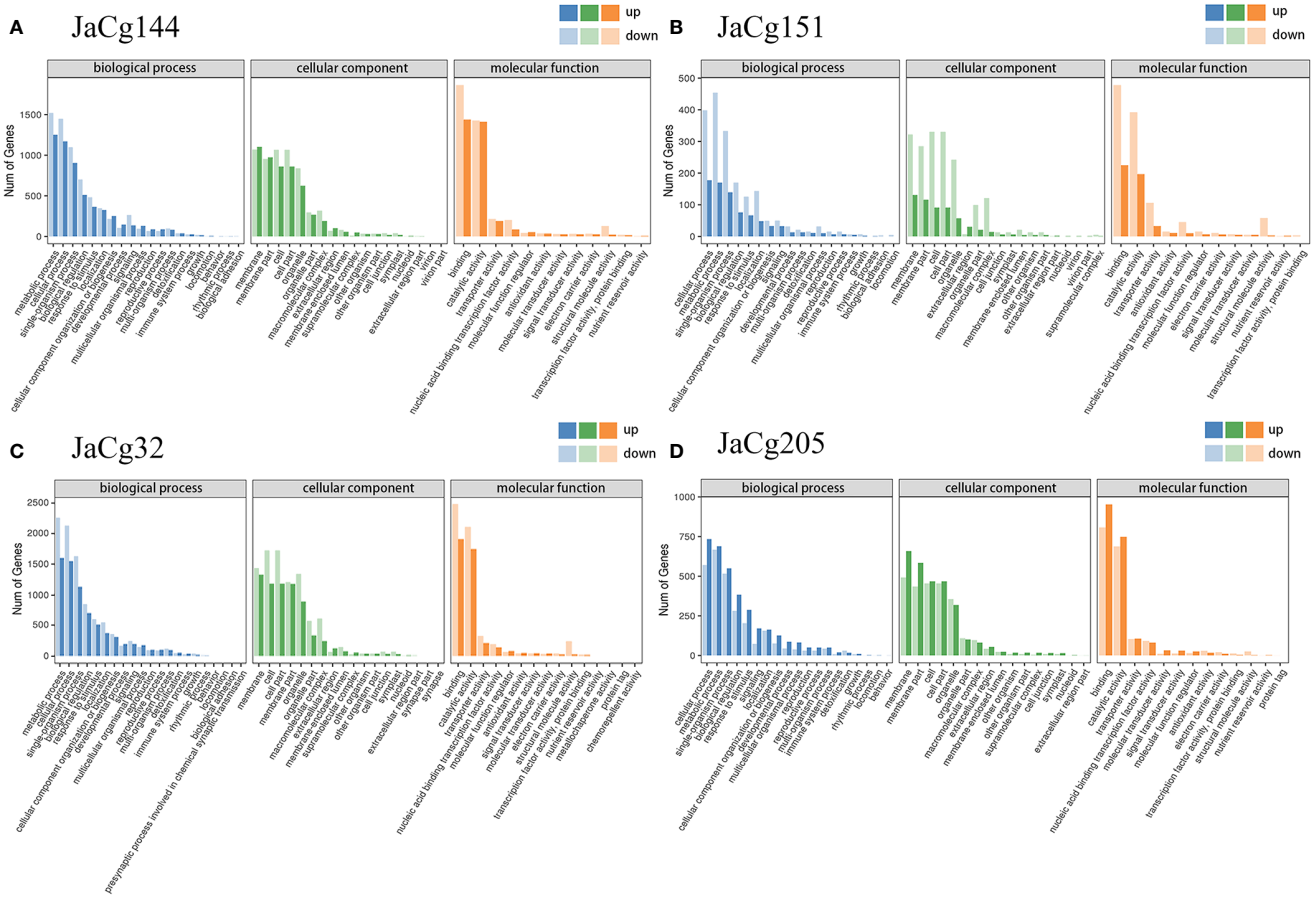
Gene ontology (GO) enrichment of the DEGs in JaCg144 **(A)**, JaCg151 **(B)**, JaCg32 **(C)** and JaCg205 **(D)** inoculation. The ordinate represents the numbers of DEGs involved in the corresponding pathways.

Ten DEGs [including BMK_Unigene_237884 (bZIP transcription factor) BMK_Unigene_234602 (Flavonoid biosynthesis), BMK_Unigene_232614 (Terpenoid backbone biosynthesis), BMK_Unigene_085590 (Response to Cd ion), BMK_Unigene_169147 (Cutin, suberine and wax biosynthesis), BMK_Unigene_170229 (ABC transporters), BMK_Unigene_160407 (Phenylpropanoid biosynthesis) and BMK_Unigene_173019 (Pentose and glucuronate interconversions)], BMK_Unigene_007177 (Oxidative phosphorylation; Photosynthesis) and BMK_ Unigene_086418 (Photosynthesis) were selected to validate the RNA-Seq using qRT-PCR analyses (**Figure 11**). The expression fold changes of these genes in *P. massoniana* were similar when comparing RNA-Seq with qRT-PCR (**Figure S4**).

### 3.9 GO and KEGG functional enrichment analyses of DEGs

To further determine the mediated effect of different ecotypes of *C. geophilum* on the *P. massoniana* to Cd stress, DEGs were applied to the GO and KEGG pathway enrichment analyses. GO enrichment analysis showed that the DEGs of mycorrhizal seedlings were significantly concentrated in biological processes, cellular components, and molecular functions (**Figure 11**). In biological processes, the most enriched GO terms were cellular process (GO:0009987), metabolic process (GO:0008152), single organism process (GO:0044699), biological regulation (GO:0065007) and response to stimulus (GO:0050896). In addition, compared to JaCg144 and JaCg205 mycorrhizal seedlings, JaCg205 mycorrhizal seedlings significantly enriched more down-regulated DEGs in the “detoxification” terms, indicating that it has poor detoxification ability to respond to Cd stress. In cell components, the unigenes were enriched to 18 terms, mostly in the cell part (G0:0044464), cell (GO:0005623), membrane (GO:0016020), membrane parts (GO:0044425) and organelle (GO:0043226). In terms of molecular function, DEG is significantly enriched to a total of 12-16 terms, mainly in binding (GO:0005488), catalytic activity (GO:0003824), and transporter activity (GO:0005215). In addition, under Cd stress, 49 DEGs were enriched in antioxidant activity terms in 4 mycorrhizal seedlings, indicating that antioxidant activity may play a crucial role in the response of mycorrhizal seedlings to Cd stress.

**Figure 12:**
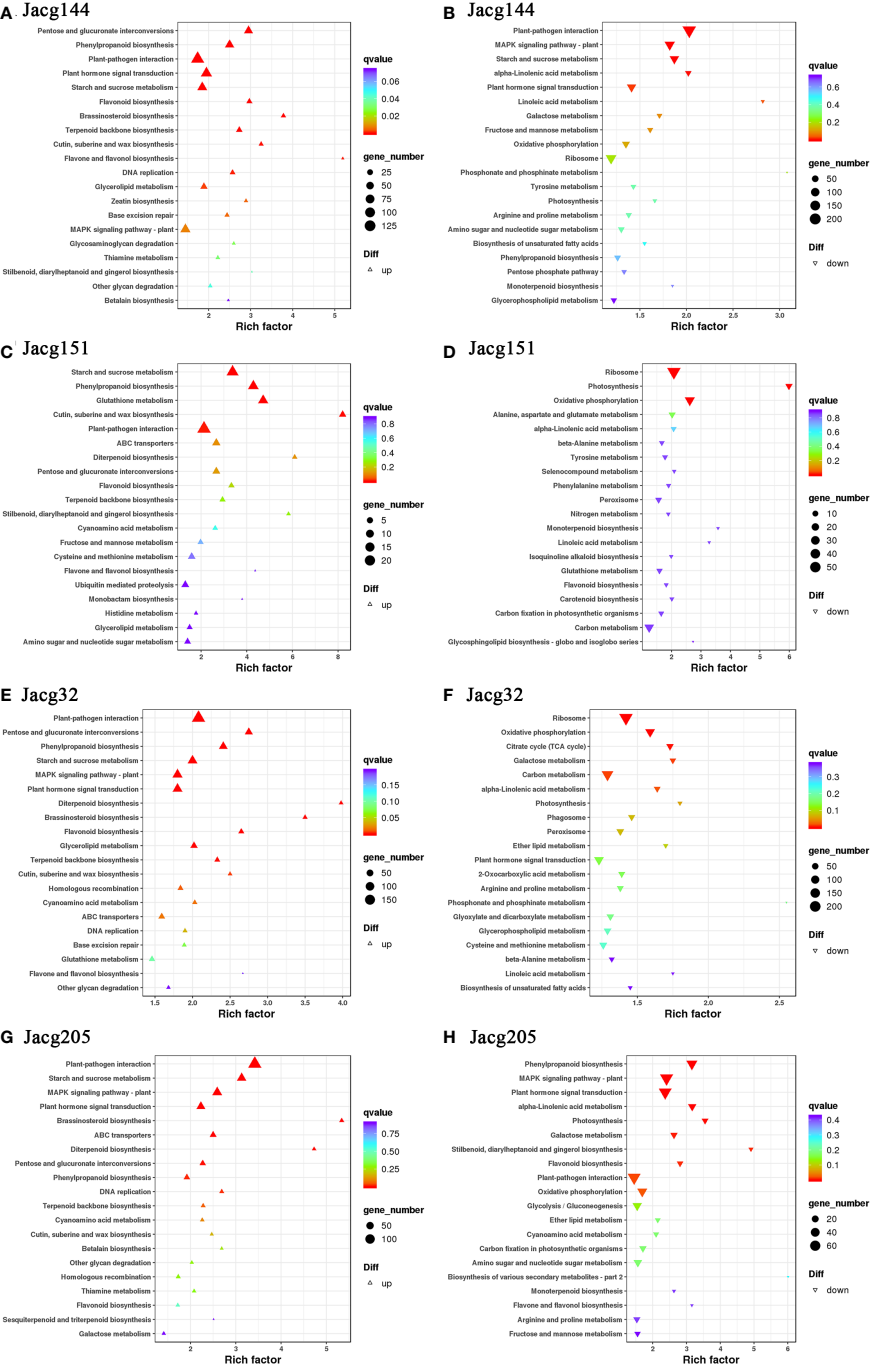
KEGG pathway enrichment analysis of DEGs. KEGG pathways of *P. massoniana* plants inoculated JaCg144 (JaCg144 CK_vs_JaCg144 Cd) up-regulated **(A)** and down-regulated genes **(B)**. KEGG pathways of *P. massoniana* plants inoculated JaCg151 (JaCg151CK_vs_JaCg151Cd) up-regulated **(C)** and down-regulated genes **(D)**. KEGG pathways of *P. massoniana* plants inoculated JaCg32 (JaCg32CK_vs_JaCg32Cd) up-regulated **(E)** and down-regulated genes **(F)**. KEGG pathways of *P. massoniana* plants inoculated JaCg205 (JaCg205 CK_vs_JaCg205 Cd) up-regulated **(G)** and down-regulated genes **(H)**. Each symbol (circle and triangle) in the figure represents a KEGG pathway, the ordinate indicates the name of the pathway, and the abscissa is the enrichment factor. The size of the symbol represents the numbers of DEGs involved in the corresponding pathways.

When analyzing the top 20 KEGG significantly enrichment pathways, it could be shown that 4 mycorrhizal seedlings enriched the pathways of “plant-pathogen interaction”, “starch and sucrose metabolism”, “oxidative phosphorylation”, “photosynthesis”, “phenylpropanoid biosynthesis”, “ABC transporters”, “pentose and glucurnate interconversions”, “flavonoid biosynthesis”, “terpenoid backbone biosynthesis” and “cutin, suberine and wax biosynthesis” (**Figure 12**). Moreover, “plant hormone signal transduction”, “MAPK signaling pathway-Plant”, “DNA replication”, “monoterpenoid biosynthesis” and “Diterpenoid biosynthesis pathways” were significantly enriched in most mycorrhizal seedlings.

It is worth noting that “the ABC transporters”, “pentose and glucuronate interconversions”, “terpenoid backbone biosynthesis”, and “cutin, suberine and wax biosynthesis” pathways of the four mycorrhizal seedlings were significantly up-regulated, which indicates that *C. geophilum* was able to enhance the tolerance of *P. massoniana* to Cd stress by promoting lipid and carbohydrate synthesis and metabolism. Importantly, the “flavone and flavonol biosynthesis” pathway was significantly up-regulated in JaCg144, JaCg151 and JaCg32 mycorrhizal seedlings, while DEGs in JaCg205 mycorrhizal seedlings were significantly down-regulated in this pathway, indicating that this pathway may be a key pathway for mycorrhizal resistance to Cd stress.

## 4 Discussion

Due to the rapid development of industrial activities, the concentration of Cd in soil and water has steadily increased (Clemens et al., 2013). Cd in the environment is a highly toxic HM for most organisms, and can be absorbed and accumulated by plants and animals, ultimately entering the human body through the food chain (Rizwan et al., 2016). Phytoremediation has been shown to be a cost-effective and environmentally-friendly technology to remediate HM polluted mine soils (Tangahu et al., 2011; Oh et al., 2014; Tang et al., 2019). Cd contaminated tailings are usually located in mountains and thus afforestation with woody plants is suitable to remediate mine tailings (Liu et al., 2020). *P. massoniana* is one of the potential tree species of afforestation in mine tailing area due to their potential applicability in the phytoremediation (Wen et al., 2016; Yu et al., 2020).

At present, ECMF assisted phytoremediation of HM contaminated soil has been shown to be an efficient and clean remediation method, and has increasingly been used for remediation of HM contaminated soil (Colpaert et al., 2011; Sousa et al., 2011; Heinonsalo et al., 2015; Chot and Reddy, 2022; Oladoye et al., 2022). However, previous studies have shown that not all fungal species were able to effectively maintain the adaptability of host plants under HM stress (Kushwaha et al., 2015; Ayangbenro and Babalola, 2017; Chot and Reddy, 2022).It is interesting that most studies have explored the impact of mycorrhizal fungi on host plant metal tolerance mainly through the study of tolerant strains (Adriaensen et al., 2006; Colpaert et al., 2011; Ma et al., 2014; Heinonsalo et al., 2015), while strains without or little tolerance to HMs tolerance have received little attention. For example, tolerant ecotypes may act as a better filter than non-tolerant ecotypes because the former more strongly prevent metals to transfer to their host (Colpaert et al., 2011). The use of such a metal-tolerant *Suillus* species has been shown to be a promising strategy to develop tools for reclamation of metal-contaminated and disturbed soils (Colpaert et al., 2011). Fungal survival in toxic substrates was shown to be an important condition for mycorrhizal protection of host plants growing on HM containing soils (Colpaert, 2008). The adaptation of trees to polluted soil largely depends on the metal adaptation potential of their associated mycorrhizal fungi (Meharg and Cairney, 1999). In summary, fungi that have the ability to tolerate HMs were able to help host plants grow in HM contaminated soil. However, is it true that the non-tolerant ecotypic strains do not have this ability?

Therefore, this experiment evaluated the Cd tolerance of 10 ecotypes of *C. geophilum* through a membership function, and then selected strains with different metal tolerance to inoculate with *P. massoniana* seedlings. After three months of cultivation under Cd stress, plant growth, root system, antioxidant enzyme activity, and Cd content in the shoots and roots were measured. Based on the membership function method, the Cd tolerance of each mycorrhizal seedling was evaluated. Surprisingly, the order of resistance to Cd stress of four different ecotypic mycorrhizal *P. massoniana* was: JaCg144 > JaCg151 > JaCg32 > JaCg205 (**Table 1**), and there was no significant correlation between the Cd tolerance of the strains and inoculated *P. massoniana* seedlings (**Table 3**). The Cd tolerance of *C. geophilum* did not affect its regulation of the host plant’s Cd tolerance performance. Moreover, it is interesting to note that JaCg144 with the weakest Cd tolerance had the strongest promoting ability on the Cd tolerance of *P. massoniana*. This indicates that ECMF did not mainly rely on their own Cd precipitation, metal exclusion, chelation and cell-wall binding etc. to alleviate the toxic effects of HMs on plants (Turnau et al., 2001; Luo et al., 2014), but mainly promoted the host plant’s tolerance to HM stress by regulating plant growth, physiology, and defense systems.

Cd contamination in soil affects plant growth not only by reducing nutrient and water availability to plants, but inhibiting photosynthesis due to chlorophyll degradation or reduced biosynthesis (**Figure 5**) (Shi et al., 2010; Amna et al., 2015). ECMF could enhanced the survival and growth of host plants under stressful environments by increasing mineral acquisition and plant development (Willis et al., 2013; Kong et al., 2020). At high levels of Cd, our result showed that inoculation with 4 ecotypes of *C. geophilum* significantly increased the fresh and dry weight of *P. massoniana*, and JaCg 144 which was a non-tolerant ecotypic strains displaying the strongest growth promotion of plants in Cd-contaminated and non-contaminated soil (**Figure 4**). This improvement can be explained by the greater root structure, and subsequent increased water and nutrient supplies to increase plant biomass production under Cd contaminated conditions, thereby mitigating the toxic effects of HMs on the plant (Ma et al., 2014; Sousa et al., 2011; Zong et al., 2015; Sun et al., 2022). Meanwhile, we observed that Cd significantly reduced the Pn, Gs, Tr and chlorophyll content of NM seedlings, but inoculation with *C. geophilum* significantly increased photosynthesis of mycorrhizal seedlings (**Figure 5**), which is consistent with the study by Liu et al. (Liu et al., 2020). Therefore, we speculated that inoculation of *C. geophilum* protected the photosynthetic system of *P. massoniana*, and facilitated the colonization of *P. massoniana* in heavy mental polluted soil. Our transcriptome results also demonstrated that Cd stress significantly down-regulated the photosynthesis and oxidative phosphorylation pathway in the KEGG of *P. massoniana* mycorrhizal seedlings, indicating that Cd inhibited plant energy transport and photosynthesis (**Figure 11**). Meanwhile, the genes encoding functions correlated to starch and sucrose metabolism pathway were significantly up-regulated under inoculation by JaCg144, JaCg151, JaCg32 and JaCg205 (**Figure 11**). Starch and sucrose, as photosynthetic products, were able to provide carbon sources for plants metabolism, indicating that inoculation of *C. geophilum* under Cd stress is beneficial to the synthesis of sucrose and starch in *P. massoniana* for the storage of energy to resist Cd stress.

MDA level enable measurement of lipid peroxidation status and cell membrane damage induced by ROS production (Li et al., 2013). We observed that *C. geophilum* inhibited the accumulation of MDA in the shoot and root (**Figure 7**). It is worth noting that the content of MDA in the shoots and roots of the JaCg144 and JaCg 151 mycorrhizal seeding were the lowest under Cd stress, with a displayed ranking of: JaCg144 < JaCg151 < JaCg32 < JaCg205 (**Figure 7**). In addition, under Cd stress, compared with NM, inoculation of *C. geophilum* significantly increased POD activity in the shoot and root of *P. massoniana* (**Figures 9E, F**). Meanwhile, KEGG enrichment analysis of DEGs showed that genes encoding phenylpropanoid biosynthesis and flavonoid biosynthesis pathways were significantly up-regulated in four *C. geophilum*-mycorrhizal seedlings (**Figure 11**). Steinkellner et al. (2007) and Agati et al. (2011) reported that flavonoid can be used as a regulatory signal molecule in the process of mycorrhizal functional symbiosis, participating in the oxidative stress response, eliminating the oxidative free radicals in cells, and improving the tolerance of plants to abiotic stress. This suggests that mitigating the lipid peroxidation status and cell membrane damage to enhance antioxidant defense are important mechanism of no-tolerant and low-tolerant ecotypic *C. geophilum* (JaCg144 and JaCg151) to enhance host plant resistance to Cd stress.

Evidences have shown that assistance by ECMF could be employed as phytomanagement to increase the phytoremediation efficiency of host plants (Kushwaha et al., 2015; Zong et al., 2015; Liu et al., 2020; Sun et al., 2022). Our study found that different *C. geophilum* significantly increased the concentrations of Cd in the roots of *P. massoniana*, but showed no effects on the shoot Cd concentrations (**Figure 10**). The concentration of HMs in plants depends on the uptake as well as the biomass, and the concentration of HMs in plants will decrease through the diluting effect of plant growth (Tang et al., 2019). Thus, it is not reasonable to determine HM uptake by plants only through the metal concentrations in plants. The success of phytoremediation greatly relies on the capacity of plants to take up HMs from soils. In this study, the inoculation of *C. geophilum* enhanced Cd accumulation amounts in shoots and roots of *P. massoniana* (**Figure 10**). This suggested that the ECMF-inoculated *P. massoniana* was able to take up higher Cd from soil than non-mycorrhizal plant. Tang et al. (Tang et al., 2019) reported that ECMF inoculation promoted the accumulation of Cu and Cd by *P. thunbergii*. Yu et al. (2020) also found that inoculation with ECMF increased the HM concentrations in the roots of *P. massoniana*. The reasons that ECMF promote plant uptake of HMs might have a number of reasons. This ectomycorrhizal network has been shown to expand the root area and the mycelium capillaries produced by ECMF enhanced the accumulation of HMs from soil to rhizosphere, which could then be easily absorbed into plant root cells by large hyphae (Tang et al., 2019; Liu et al., 2020). In mycorrhiza plants, HMs were shown to be transported to the Hartig net for loading into root cells (Liu et al., 2020). Thus, ECMF usually inhibited the transfer of HMs from roots to shoots (Meharg and Cairney, 1999; Colpaert et al., 2011; Luo et al., 2014; Cundy et al., 2016; Yang et al., 2016; Wen et al., 2017; Liu et al., 2020; Liu et al., 2020). In addition, our data showed that the TF of *C. geophilum*-mycorrhizal seedlings was lower than in NM, which supported the view that ectomycorrhizal plants are able to accumulate more Cd in roots than shoots (**Figure 10D**). It was also found in our study that the inoculation with different *C. geophilum* has differential effects on the absorption of HMs by *P. massoniana*. Among the different *C. geophilum*-mycorrhizal seedlings, JaCg151 and JaCg 144 displayed the best promotion effects (**Figure 10**). Overall, our results suggested that inoculation with *C. geophilum* increases the absorbtion of Cd by *P. massoniana*, thereby improving the efficiency of phytoremedaition. Therefore, the symbiosis between *C. geophilum* and *P. massoniana* shows the great potential for phytoremediation of Cd polluted soil.

## 5 Conclusion

In conclusion, we found that *C. geophilum* is one of candidate ECMF for the revegetation of soil containing a high content of Cd. The Cd tolerance of *C. geophilum* did not affect its regulation of the host plant’s Cd tolerance performance. *C. geophilum* which are no-tolerant and low-tolerant ecotype also had the strong promoting effect on the Cd tolerance of *P. massoniana*, while significantly increasing the accumulation of Cd in roots of seedlings. Hence, we speculated that ECMF did not mainly rely on their own Cd precipitation, metal exclusion, chelation and cell-wall binding etc. to alleviate the toxic effects of Cd on plants, but mainly promoted the host plant’s tolerance to Cd stress by regulating plant growth, physiology, and defense systems. This research demonstrated that non-tolerant ECMF can be used as potential inoculation fungal resources for future phytoremediation, and provides a more feasible strategy for ECMF assisted phytoremediation.

## Data availability statement

The datasets presented in this study can be found in online repositories. The names of the repository/repositories and accession number(s) can be found in the article/**Supplementary Material**.

## Author contributions

TZ: Methodology, Supervision, Writing – original draft, Writing – review & editing. WP: Conceptualization, Data curation, Software, Writing – review & editing. TY: Methodology, Software, Writing – review & editing. PZ: Methodology, Writing – review & editing. JH: Software, Writing – review & editing. CR: Supervision, Writing – review & editing. WY: Funding acquisition, Writing – review & editing. CL: Supervision, Writing – review & editing.
