# Supplementary figures and images for: Metal-non-tolerant ecotypes of ectomycorrhizal fungi can protect plants from cadmium pollution
Source: Front Plant Sci. 2023 Dec 6;14:1301791. doi: 10.3389/fpls.2023.1301791 (PMC10731278; doi:10.3389/fpls.2023.1301791)

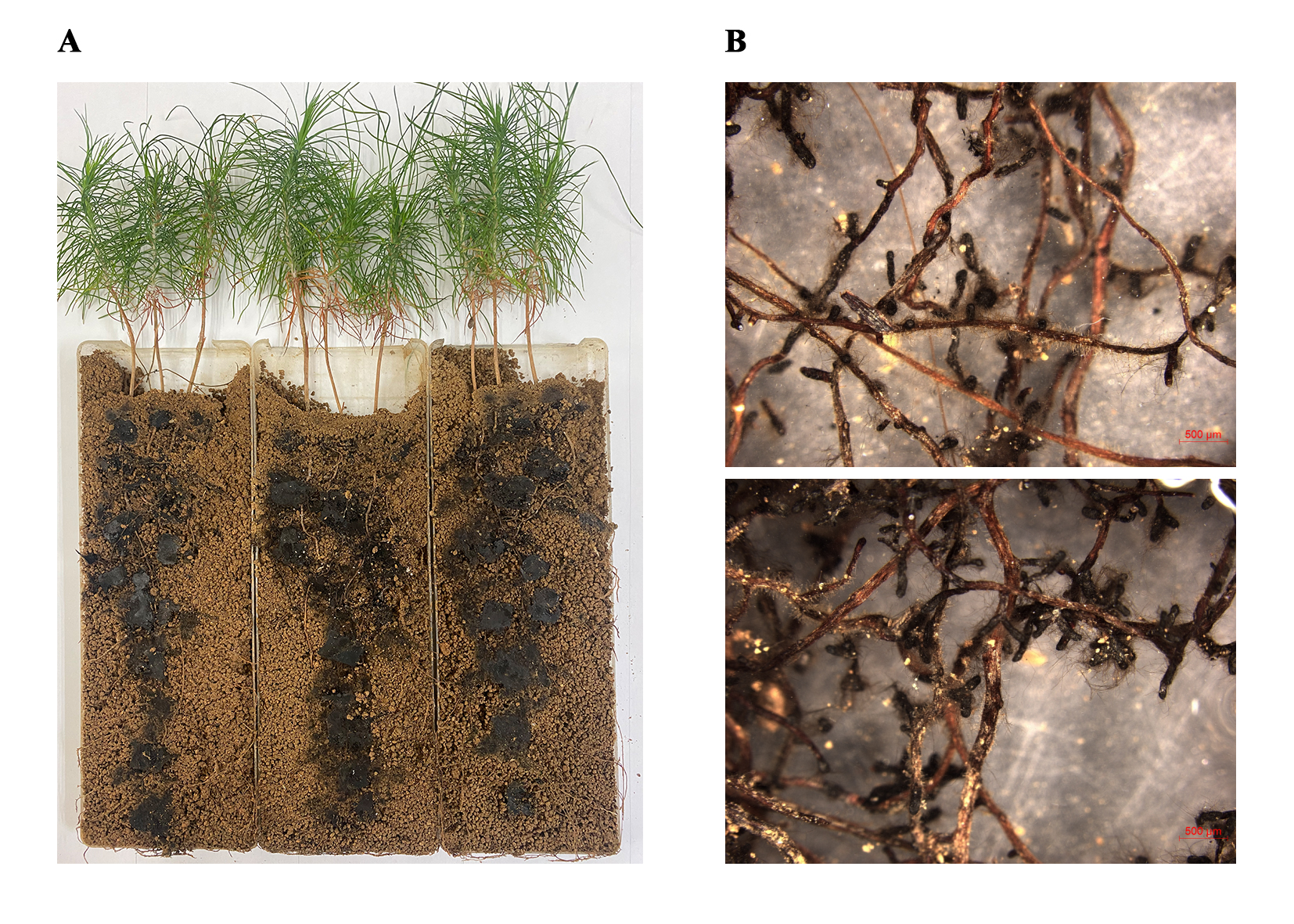

Supplement: Supplementary file 1 [file Image_1.tif]

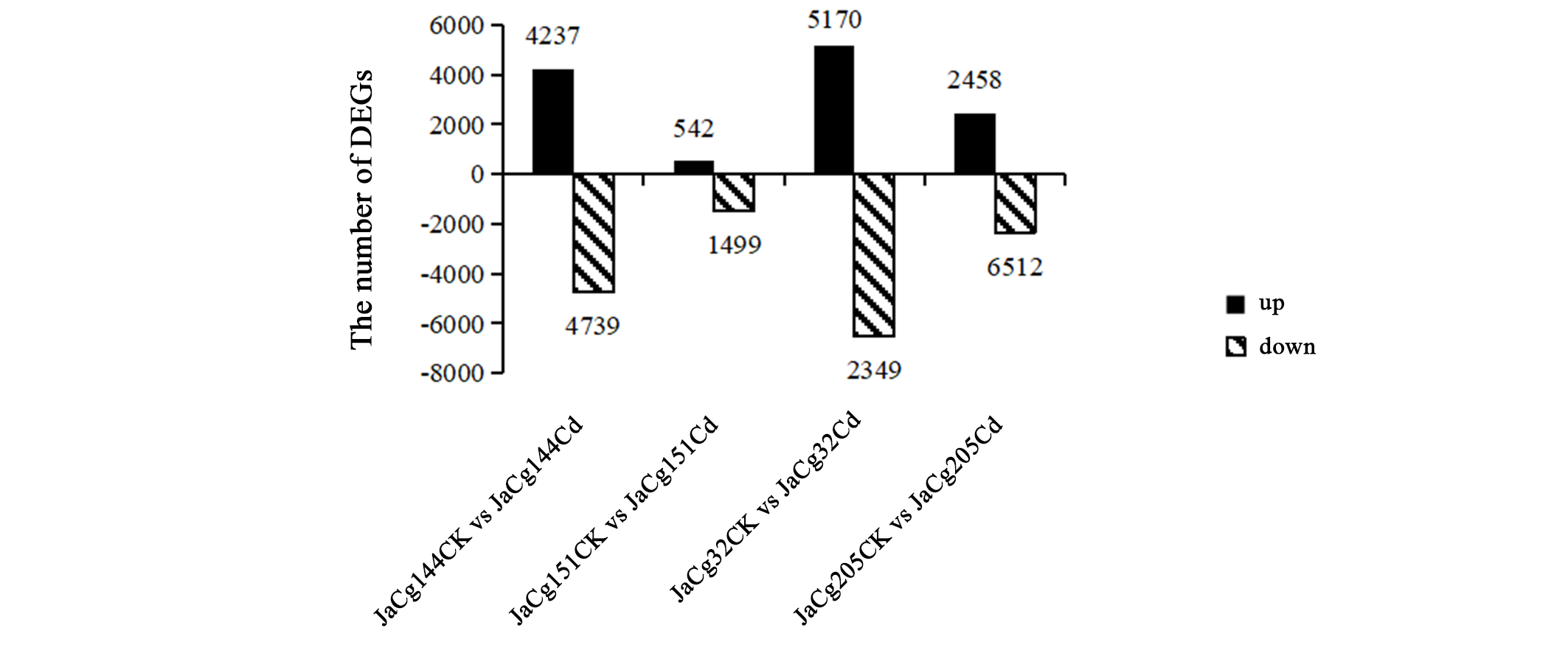

Supplement: Supplementary file 2 [file Image_2.tif]

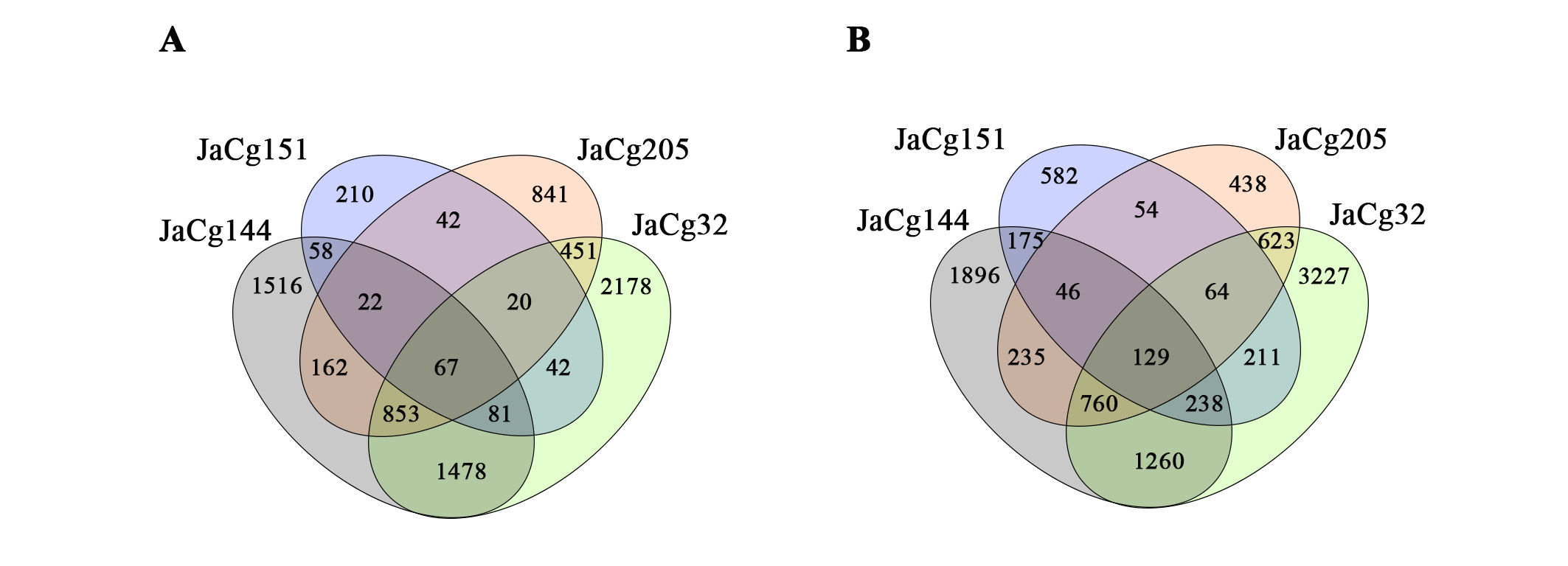

Supplement: Supplementary file 3 [file Image_3.tif]

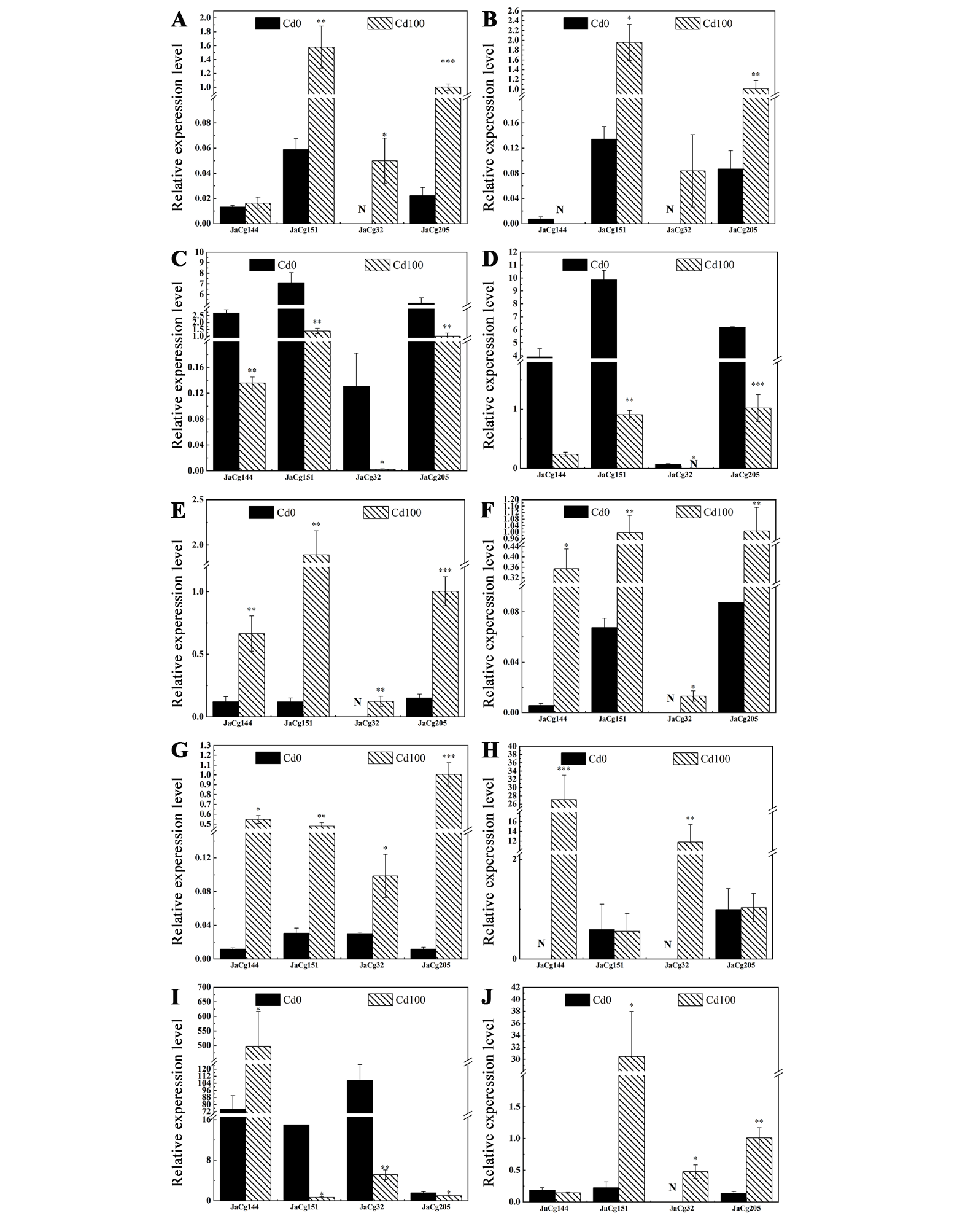

Supplement: Supplementary file 4 [file Image_4.tif]
